# Reply to Chen et al.: Coarse simulations overestimate the distance to recover NO–NO_2_–O_3_ photochemical steady state in fresh NO_x_ plumes

**DOI:** 10.1073/pnas.2425976122

**Published:** 2025-02-10

**Authors:** Daniel J. Varon, Dylan Jervis, Sudhanshu Pandey, Sebastian L. Gallardo, Nicholas Balasus, Laura Hyesung Yang, Daniel J. Jacob

**Affiliations:** ^a^Harvard University, School of Engineering and Applied Sciences, Cambridge, MA 02138; ^b^GHGSat, Inc., Montréal, QC H2W 1Y5, Canada; ^c^Jet Propulsion Laboratory, California Institute of Technology, Pasadena, CA 91109; ^d^Centro Atomico Bariloche, Bariloche R8402AGP, Argentina

We thank Chen et al. (1) for their comment on our paper (2). The comment focuses on the departure of the NO_x_/NO_2_ ratio from ambient NO–NO_2_–O_3_ photochemical steady state (PSS) in fresh power plant plumes due to ozone depletion by reaction with emitted NO. This departure will cause a low bias in the NO_x_ emission estimate if the NO_x_/NO_2_ ratio from ambient PSS is used to convert the NO_2_ flux inferred from the observations by the cross-sectional flux method, as we did in our paper. Indeed, we show in figure 2 of our paper that the NO_2_ flux increases downwind of the smokestack (implying a decrease in the NO_x_/NO_2_ ratio) but we also find that the increase levels off about 2 km downwind, implying that ozone has recovered from its depletion by the freshly emitted NO and that the ambient NO_x_/NO_2_ PSS assumption is applicable. Our estimate of the NO_x_ flux is based on the NO_2_ flux at 2 to 3 km downwind of the source (our equation 7). We also point out that this simple relaxation to ambient steady state is not always observed (e.g., for the Bridger power plant).

Chen et al. find in their WRF-CMAQ simulations with 1-km resolution that relaxation to ambient PSS may not be achieved even 30 km downwind of the source. However, such coarse model resolution is not appropriate to simulate the near-field dispersion of a point source. As seen in figure 1 of our paper, the plumes would not be properly resolved in a model with 1-km resolution. Modeling plume dispersion from a point source and the associated NO–NO_2_–O_3_ kinetics requires a large-eddy simulation with resolution ~100 m. Sykes et al. (3) (cited in our paper) used such a simulation to determine the distance downwind of the source for relaxation to ambient PSS. They found a distance of 1 to 2 km (their figure 4), consistent with our results. The WRF-CMAQ simulation with 1-km resolution underestimates the rate of plume dispersion and entrainment of ambient ozone because it does not account for turbulent horizontal diffusion (parameterization of small eddies), which dominates plume dispersion at sub-km scales. Relaxation to ambient PSS would happen much faster than in the WRF-CMAQ simulation.

This being said, there is substantial uncertainty in the NO_x_/NO_2_ ratio from ambient PSS. The ratio depends on ozone concentration, solar radiation, and temperature. We used a ratio of 1.38 ± 0.10 from Beirle et al. (4), but the uncertainty may be larger. There are however other factors of error in our satellite-based inferences of NO_x_ fluxes including uncertainties in the NO_2_ retrieval and in the representative wind speed. We characterized our overall error by comparison to independent estimates (table 1 of our paper) and found an 11% low bias that we attributed tentatively to incomplete recovery of ambient PSS. The analysis of Chen et al. seriously overestimates the distance required for the NO_x_/NO_2_ ratio to relax to ambient PSS and we see no reason to revise our conclusions.
